# Predicting Early Post-stroke Aphasia Outcome From Initial Aphasia Severity

**DOI:** 10.3389/fneur.2020.00120

**Published:** 2020-02-21

**Authors:** Alberto Osa García, Simona Maria Brambati, Amélie Brisebois, Marianne Désilets-Barnabé, Bérengère Houzé, Christophe Bedetti, Elizabeth Rochon, Carol Leonard, Alex Desautels, Karine Marcotte

**Affiliations:** ^1^Centre de Recherche du Centre Intégré Universitaire de Santé et de Services Sociaux du Nord-de-l'Île-de-Montréal, Montreal, QC, Canada; ^2^École d'Orthophonie et d'Audiologie, Université de Montréal, Montreal, QC, Canada; ^3^Centre de Recherche de l'Institut Universitaire de Gériatrie de Montréal, Montreal, QC, Canada; ^4^Département de Psychologie, Université de Montréal, Montreal, QC, Canada; ^5^Department of Speech-Language Pathology, University of Toronto, Toronto, ON, Canada; ^6^Toronto Rehabilitation Institute, Toronto, ON, Canada; ^7^Heart and Stroke Foundation, Canadian Partnership for Stroke Recovery, Ottawa, ON, Canada; ^8^Rehabilitation Sciences Institute, University of Toronto, Toronto, ON, Canada; ^9^School of Rehabilitation Sciences, University of Ottawa, Ottawa, ON, Canada; ^10^Département de Neurosciences, Université de Montréal, Montreal, QC, Canada; ^11^Centre d'Études Avancées en Médecine du Sommeil, Hôpital du Sacré-Cœur de Montréal, Montreal, QC, Canada

**Keywords:** aphasia, white matter (WM), dMRI (diffusion magnetic resonance imaging), stroke, early recovery, linguistic assessment

## Abstract

**Background:** The greatest degree of language recovery in post-stroke aphasia takes place within the first weeks. Aphasia severity and lesion measures have been shown to be good predictors of long-term outcomes. However, little is known about their implications in early spontaneous recovery. The present study sought to determine which factors better predict early language outcomes in individuals with post-stroke aphasia.

**Methods:** Twenty individuals with post-stroke aphasia were assessed <72 h (acute) and 10–14 days (subacute) after stroke onset. We developed a composite score (CS) consisting of several linguistic sub-tests: repetition, oral comprehension and naming. Lesion volume, lesion load and diffusion measures [fractional anisotropy (FA) and axial diffusivity (AD)] from both arcuate fasciculi (AF) were also extracted using MRI scans performed at the same time points. A series of regression analyses were performed to predict the CS at the second assessment.

**Results:** Among the diffusion measures, only FA from right AF was found to be a significant predictor of early subacute aphasia outcome. However, when combined in two hierarchical models with FA, age and either lesion load or lesion size, the initial aphasia severity was found to account for most of the variance (*R*^2^ = 0.678), similarly to the complete models (*R*^2^ = 0.703 and *R*^2^ = 0.73, respectively).

**Conclusions:** Initial aphasia severity was the best predictor of early post-stroke aphasia outcome, whereas lesion measures, though highly correlated, show less influence on the prediction model. We suggest that factors predicting early recovery may differ from those involved in long-term recovery.

## Introduction

Aphasia represents one of the most devastating cognitive consequences of a stroke. It is associated with higher levels of anger, loneliness, social isolation, and greater difficulties in resuming daily life activities (e.g., return to work) (1). The resulting impairments can partially recover in the days, weeks, or months after a stroke (2), but the degree of recovery varies widely across individuals (3–5). To date, the degree of recovery has been primarily associated with three kinds of factors (6): demographic variables (such as age or education) (4), lesion-related variables (such as lesion size and lesion location) (7, 8), and clinical variables (including the type and severity of aphasia, and also treatment provided to the patient) (9). While demographic variables have a weak association with long-term outcomes (10), lesion-related factors have been shown to have a strong relationship with long-term recovery (6, 11). However, clinical variables remain the most widely used measures for clinicians to gain insight into the patient's clinical progression (12). Current research focuses on investigating which are the most reliable factors that enable clinicians to predict long-term outcomes and that help predict recovery.

Among the clinical variables, initial aphasia severity seems to be one of the best predictors of aphasia outcome (4, 13, 14). For instance, Kertesz and McCabe showed that the initial Aphasia Quotient [AQ, aphasia severity scale from the Western Aphasia Battery (15), henceforth referred to as WAB] was a good predictor of aphasia recovery at 6-and 12-months, while age or sex did not improve prognosis accuracy (16). More recently, Lazar and colleagues proposed a modified version of the AQ for acute stroke assessment (mean = 2.1 days) (13). Their mean composite score was composed of the comprehension, repetition and naming sections of the WAB, having all sections equal weight on the final score. Using this modified AQ, they reported that initial severity was a good predictor of recovery during the first 90 days post-stroke. Although the results were clear, this study evaluated patients with only mild to moderate aphasia, which neglects those patients with more severe language deficits in which recovery results are more difficult to capture. A recent study found evidence that the interaction between severity and other variables may be different in patients with more severe aphasia (17). Inclusion of patients with severe aphasia entails more difficulty in the analysis of data, but is necessary to picture a more realistic and clinically relevant scenario (12). Furthermore, another gap in the literature is the study of the spontaneous recovery, scarcely studied in the weeks after stroke onset (3, 18, 19), and impossible to analyze in longitudinal studies due to the effect of therapy and rehabilitation. Recently, Wilson and colleagues described the evolution of aphasia during the first 2 weeks after a stroke, and explored how language improves promptly in different modalities within the first week post-stroke (20). However, no measures were taken to assess the biomarkers that might predict this recovery.

As for lesion-related factors, they are also broadly used to predict aphasia outcomes. Although lesion size has been shown to be a good predictor of stroke and aphasia outcomes (7, 21, 22), the study of specific damaged structures has recently been determined to be a more accurate index for specific impairments. Because most patients with post-stroke aphasia have damage near/in the middle cerebral artery (23), lesions to specific structures in this territory have been linked to aphasia symptoms. For instance, the superior temporal gyrus, the pars opercularis of the inferior frontal gyrus, the anterior insula and the supramarginal gyrus are among the areas most frequently related to aphasia symptoms (24). However, contemporary frameworks of language processing consider language functions to be a result of processing cores working in an interconnected network. This functional network is supported by pathway structures linking the areas of processing, i.e., the white matter bundles. Therefore, if white matter structures are important to establish linguistic abilities, they may be good candidates to support aphasia recovery (25).

Among all the white matter structures in the brain, probably the one that is the most studied in relation to language is the arcuate fasciculus (AF) (26, 27). This fiber bundle, which connects areas from the temporal, parietal and frontal cortical areas through its three segments (23), has been linked to several language functions, from speech-in-noise perception to syntax processing. Researchers have used diffusion magnetic resonance imaging (dMRI) measures to assess the influence of the lesioned AF in the language breakdown, either through the integrity of its structure (28–30) or through its properties. Other approaches include combinations of gray and white matter (31, 32), or quantitative measures of the spared white matter in the contralesional hemisphere (33, 34). Interestingly, some studies have found a relation between diffusion measures of different white matter fiber bundles and language outcomes in the early phases of post-stroke aphasia (35, 36). However, there is a lack of evidence regarding the changes in white matter and how this is related to early and spontaneous recovery from aphasia.

In this study, we intended to explore outcomes of aphasia in the first 2 weeks after stroke onset. We also intended to elucidate which factors, either related to the lesion characteristics or the preserved language skills, are accurate predictors of these outcomes in patients at the beginning of their subacute phase, before having received any therapy. To our knowledge, no previous study has evaluated the degree of improvement between the acute and sub-acute phase using analyses that combine more than one language ability and neuroimaging measures. This work could provide new information that can be used to improve the prediction of aphasia recovery and the planification of rehabilitation of patients in the long-term. Based on previous evidence (13, 20), we hypothesized that initial severity will predict the early recovery, but only partially given that the dynamics of recovery are more unstable in this phase than in the phases more commonly reported in the literature (e.g., at 3, 6 months post-onset). We also predicted that there is a relationship between the diffusion measures from the arcuate fasciculus (bilaterally), given its proven importance as a predictor for language abilities in other studies (30, 34, 37), and the early outcomes 2 weeks after onset.

## Materials and Methods

### Participants

Twenty participants took part in this study (5 women; mean age: 71.6 ± 12.45 years; mean education: 10.05 ± 5.04). Participants presented with aphasia due to a first single ischemic stroke in the left middle cerebral artery. All participants were diagnosed by a neurologist at the Stroke Unit at Hôpital du Sacré-Coeur de Montréal and screened for eligibility. The aphasia severity rating scale from the Boston Diagnostic Aphasia Examination test (38) was used to obtain an initial severity score. Initial language assessments took place within the first 72 h (mean = 2.3 days) after stroke onset, and the follow-up took place 7–15 days later (mean = 10.55 days). Clinical and sociodemographic information of the entire sample are presented in Table 1. All participants were fluent speakers of French or English before stroke and completed their evaluation either in French (*n* = 19) or in English (*n* = 1). Five participants were monolinguals (Canadian French only), thirteen were bilinguals (12 spoke Canadian French and English and 1 spoke English and Dutch) and two spoke three languages or more (Canadian French and other languages). Exclusion criteria included a history of major psychiatric disorder(s), learning disabilities, severe perceptual deficits, additional neurological diagnoses or left-handedness. No participant presented with pronounced subcortical arteriosclerosis. The study was approved by the ethics review board (Project #MP-32-2018-1478) of the research center of the Centre intégré universitaire de santé et de services du Nord-de-l'Île-de-Montréal, in the Hôpital Sacré Coeur de Montreal. Written informed consent was obtained from all participants.

**Table 1:** Participants' sociodemographic and clinical information.

	**Sex**	**Age**	**Educ.** ** (years)**	**Initial** ** NIHSS** Score^*^^§^	**rTPA**	**Aphasia** ** type**	**Severity** ** (BDAE Scale)**	**Lesion location**				**Lesion** ** size** ** (mL)**	**Initial** ** assessment** ** (# days** ** post-stroke)**	**Follow-up** ** assessment** ** (# days** ** post-stroke)**
								**Frontal**	**Temporal**	**Parietal**	**Subcortical**			
1	M	52	9	n/a	Yes	TC mixed	Moderate to severe		X			35	1	7
2	M	74	6	9	Yes	Wernicke	Severe		X	X	X	20	3	8
3	M	61	10	6	No	Broca	Moderate to severe	X			X	12	3	11
4	M	49	9	6	No	Anomic	Mild to moderate	X		X	X	2	2	9
5	M	73	19	18	No	Wernicke	Severe		X		X	16	3	10
6	M	83	9	9	No	TC sensory	Moderate	X		X		35	3	10
7	F	73	7	n/a	No	TC sensory	Moderate	X	X	X		6	3	13
8	M	65	11	6	Yes	Anomic	Mild		X	X		12	3	14
9	M	72	15	11	Yes	TC mixed	Moderate to severe	X		X	X	1	1	9
10	M	55	11	23	Yes	TC mixed	Moderate to severe	X			X	98	2	10
11	M	87	9	6	No	Anomic	Mild	X				3	3	9
12	M	73	11	n/a	Yes	Wernicke	Moderate to severe		X	X	X	16	1	8
13	M	64	15	n/a	Yes	Conduction	Mild			X		16	1	11
14	F	95	6	1	No	Broca	Mild to moderate			X		13	2	9
15	F	60	12	7	Yes	Anomic	Mild to moderate	X	X		X	0.26	3	13
16	M	91	19	7	No	Anomic	Mild to moderate	X			X	0.10	3	15
17	F	85	16	n/a	No	TC mixed	Moderate			X		14	2	8
18	M	71	7	n/a	No	TC motor	Moderate	X		X		1	2	10
19	F	81	15	17	Yes	Anomic	Mild			X		10	2	13
20	F	68	12	n/a	Yes	Anomic	Mild	X		X		0.33	3	12

*rTPA, Recombinant tissue plasminogen activator; BDAE scale, Boston Denomination Aphasia Examination severity scale; NIHSS scale, National Institutes of Health Stroke Scale (scale of severity, from 0 to 42)*.

* *Higher values indicate higher overall severity of stroke and poorer prognosis*.

§ *The reported NIHSS is the score obtained in the emergency room by a neurologist. This assessment was not conducted for six participants (n/a)*.

### Rationale, Construction, and Scoring of the Aphasia Composite Score

Based on Lazar et al. (13) we developed a composite score (CS) adapted for the French- and English-speaking population that consisted of three subscores: comprehension, repetition and naming. For the comprehension subscore, we combined the word-sentence comprehension Task (max = 47 points) of the Montreal-Toulouse aphasia battery (MT-86) (39) and the revised (short) version of the Token Test (40) (max = 36 points), which includes oral comprehension of words, sentences and sequential commands. The repetition subscore was assessed using the repetition task [2 points for each word/non-word (*n* = 30) and 5 points for each sentence (*n* = 3), max = 75 points] of the MT-86 (39). Finally, the naming subscore consisted of the semantic fluency task (max = 25 points) of the Protocole Montréal d'Évaluation de la Communication (41) and a naming task. The test Dénomination orale d'images (DO-80) (42) (max = 80 points) was used for participants tested in French and the Boston Naming Test (BNT) (43) (max = 60 points) was used for the one participant who was tested in English, since there is no adaptation of this test currently available in English and its characteristics as naming test are the same as the BNT. Each of the three subscores was computed to a possible score of 10, so the maximum CS was equal to 30. Initial aphasia severity (CS _initial_) and sub-acute severity (CS_subacute_) were calculated for each participant, as well as their potential recovery (potential recovery = 30–CS _initial_) and their achieved recovery (achieved ΔCS = CS_subacute_-CS _initial_). A percentage of factual recovery per individual was computed as achieved recovery = (achieved ΔCS/Potential recovery).

### Neuroimaging Processing and Tractography Analyses

Participants underwent an MRI scan the same day of each language assessment. The MRI protocol was acquired using a Skyra 3T MRI scanner (Siemens Healthcare, USA) at the Radiology Department of Hôpital du Sacré-Coeur in Montreal. One high resolution 3D T1-weighted scan was acquired using a Magnetization Prepared Rapid Gradient Echo (MP-RAGE) sequence (TR = 2,200 ms, TE = 2.96 ms, TI = 900 ms, voxel size = 1 ×1 ×1 mm^3^, matrix = 256 ×256, 192 slices, flip angle = 8 degrees). A diffusion weighted imaging (DWI) series of sequences in a posterior-anterior acquisition (64 images with non-collinear diffusion gradients at *b* =1,000 s/mm^2^ with TR = 8,051 ms, TE = 86 ms, FOV = 230 mm, voxel size = 2 mm ×2 mm ×2 mm, flip angle = 90 degrees, bandwidth = 1,698 Hz; EPI factor = 67) was also acquired. In addition, two T2-weighted images at b = 0 s/mm^2^ were also acquired one in a posterior-anterior acquisition, one in an anterior-posterior acquisition to correct for distortion caused by magnetic field in homogeneities. Stroke lesions were demarcated using a semi-automated demarcation performed with *Clusterize* (44) (http://www.medizin.uni-tuebingen.de/kinder/en/research/neuroimaging/software/). Agreement between a manual segmentation and the semi-automated lesion maps obtained with *Clusterize* has been shown to be excellent in acute stroke using CT, DWI and T2 FLAIR (45). Moreover, ADC maps extracted from the DWI sequence are less sensitive to imaging artefacts (i.e., T2-shine-through) than DWI images (46) and both have high sensitivity for detecting acute ischemic stroke (47). Thus, stroke lesions were segmented with the ADC maps using *Clusterize*, and were verified and corrected by two other independent judges afterwards. Lesion size was estimated in mL. After lesion demarcation, regions of interest were extracted using FreeSurfer (https://surfer.nmr.mgh.harvard.edu) and tensors and fiber orientation maps were obtained using MRtrix3. Previous research has shown the importance of the AF for recovery from aphasia, but some studies indicate the AF in the left hemisphere is more important (30, 37), whereas others suggest the right hemisphere is relevant for recovery (34). Based on this converging evidence regarding the role of the long segment of the AF in language recovery in patients with aphasia, we extracted the fractional anisotropy (FA), the axial diffusivity (AD) and the lesion load of this fiber bundle in both hemispheres. AD was chosen over other diffusivity measures since it has been more directly related to acute post-stroke recovery in motor impairments compared to other measures (48). Lesion load was calculated from the number of voxels that were defined as AF inside the lesion size of each participant, weighted by the number the same voxels occupied by the AF in healthy participants, described in another study of our team (49).

### Statistical Analyses

First, we performed tests on the behavioral measures alone to evaluate whether there was a significant improvement of language impairment during the first 2 weeks following a stroke. Since CS_subacute_ and some of the subscores showed a non-normal distribution (a Shapiro Wilk normality test revealed the scores for comprehension_(subacute)_, repetition_(initial)_ and repetition_(subacute)_ being *P* <0.05 in all cases), we conducted a Wilcoxon signed rank test for paired-samples between CS_initial_ and CS_subacute_ and between the paired subscores, with at least one subscore having a non-normal distribution. For the other pair whose distribution was normal (naming), a paired-sample *t*-test was used. We also inspected how much of the achieved score was influenced by the potential recovery.

Second, we performed different analyses to determine which variables are the best predictors for CS_subacute_. We first performed a series of Pearson correlations to test the association between all our variables of interest with CS_subacute_. Correlation analyses were corrected at a level of significance of α = 0.01. Subsequently, to test which variables best fit an ultimate regression model, we performed several regressions analyses in different steps. In a first step, a backwards analysis was performed to determine which diffusion variables extracted from the arcuate fasciculus (i.e., FA from left AF; FA from right AF; AD from left AF; AD from right AF) was more so related to the dependent variable. The variables that were found to be significant were included in a hierarchical multivariate regression later. Two models of this hierarchical regression were tested. Both of them were computed in three blocks: in the first block, age, and initial aphasia severity were entered as control variables, or covariates (since previous research has already shown a certain capacity of prediction of both of them for later outcomes in aphasia) (6); in the second block, we introduced either lesion size (first hierarchical model) or lesion load of the left AF (second hierarchical model); in the third block, we introduced the significant diffusion variables from the first regression that we performed. Doing so, we could differentiate the contribution of the patient-related- and the different lesion-related-factors in the final prediction of the outcome.

## Results

**Figure 1:**
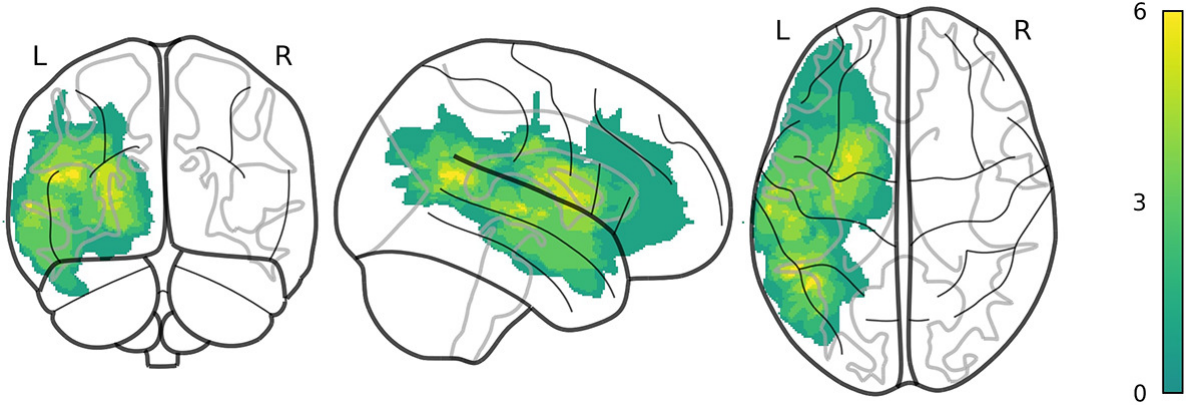
An overlay map of individual lesions of all 20 participants. Color scale indicates the number of participants with lesions in the same location.

Individual CS scores during the initial and second assessment are reported in Table 2. A lesion overlay map can be seen in Figure 1. Three participants showed a deterioration between the two assessments; the rest of the participants showed an improvement in CS scores. As a group, the mean CS_initial_ was 17.57 (*SD* = 7.55), whereas the mean CS_subacute_ was 21.68 (*SD* = 6.01). There was a significant overall improvement in language functioning during the follow-up (*Z* = 3.547, *P* <0.001). The mean improvement in CS for the whole group was 33% (*SD* = 26.9), i.e., 33% of the potential recovery was achieved on average. All three subscores (i.e., comprehension, repetition and naming) significantly improved between the initial assessment and the follow-up (Comprehension Wilcoxon signed ranks test, *Z* = 3.771, *P* <0.001; Repetition Wilcoxon signed ranks test, *Z* = −3.115, *P* = 0.002; Naming paired-sample *t*-test = −2.329, *df* = 18, *P* = 0.031). A visual comparison can be seen in the Supplementary Figure 1. Achieved ΔCS positively correlated with the potential ΔCS (*r* = 0.651, *P* = 0.002). A visual representation can be found in the Supplementary Figure 2.

**Table 2:** Individual and mean scores of each of the subscores and Composite Scores at both assessments and related recovery measures.

**Subjects**	**Naming _**initial**_**	**Repetition _**initial**_**	**Comprehension _**initial**_**	**CS** **initial^a^**	**Naming _**subacute**_**	**Repetition _**subacute**_**	**Comprehension _**subacute**_**	**CS** **subacute^b^**	**Achieved ΔCS^c^**	**Potential ΔCS^d^**
	**Acute assessment**				**Subacute assessment**				**Recovery measures**	
1	1.71	2.93	3.55	8.20	8.47	7.86	8.43	24.77	16.58	21.80
2	3.14	2.40	4.69	10.24	3.52	4.80	5.48	13.80	3.56	19.76
3	2.47	0.53	8.31	11.32	3.80	2.13	9.39	15.32	3.83	18.68
4	6.57	9.33	8.91	24.82	8.28	9.33	9.81	27.43	2.62	5.18
5	0.85	2.93	3.91	7.71	0.76	7.60	5.66	14.02	6.31	22.29
6	2.09	0	1.80	3.90	4.00	6.53	3.85	14.38	10.50	26.10
7	2.95	8.40	3.01	14.36	3.52	8.40	5.30	17.22	2.86	15.64
8	10.00	9.73	8.79	28.53	8.47	10.00	8.97	28.88	0.35	1.47
9	6.76	867	5.90	21.33	8.47	10.00	9.63	28.11	6.77	8.67
10	1.33	5.87	3.43	10.63	0.38	5.60	3.97	9.89	−0.74	19.37
11	6.66	8.27	9.27	24.21	5.33	8.93	9.69	23.96	−0.72	10.65
12	3.33	5.33	4.09	12.76	4.28	5.86	4.63	14.79	2.03	17.24
13	9.14	8.80	9.51	27.46	9.80	9.33	9.75	28.90	1.44	2.54
14	5.90	4.40	5.96	16.27	7.90	7.06	7.89	22.86	6.59	13.73
15	8.19	8.67	6.74	23.60	6.28	8.40	7.04	21.73	−1.87	6.40
16	4.57	5.20	7.65	20.09	7.71	8.66	9.45	25.07	4.99	9.91
17	7.71	7.73	0	12.30	5.61	7.86	6.80	22.38	10.08	17.70
18	5.04	7.33	6.20	18.59	5.61	8.80	7.40	21.82	3.24	11.41
19	8.57	9.07	9.15	26.79	8.66	10.00	9.03	27.70	0.91	3.21
20	6.85	10.00	9.87	26.74	9.80	933	9.63	28.78	2.04	3.26
Mean (SD)	5.19 (2.82)	6.28 (3.14)	6.04 (2.88)	17.57 (7.55)	6.03 (2.81)	7.83 (2.01)	7.59 (2.11)	21.68 (6.01)	4.10 (4.31)	12.43 (7.55)

a *CS_initial_ = (Naming_initial_ + Comprehension_initial_ + Repetition_initial_)*.

b *CS_subacute_ = (Naming_subacute_ + Comprehension_subacute_ + Repetition_subacute_)*.

c *Achieved ΔCS = (CS_subacute_ – CS _initial_)*.

d *Potential ΔCS = (30- CS _initial)_*.

Only one model was significant as a result of the backwards regression analysis that used the diffusion variables and CS_subacute_ as dependent variable. The model included FA from right AF (rFA) and AD from left AF after elimination of the less contributing variables (*R*^2^ = 0.282). From these two variables, only rFA had a significant beta coefficient (β = 0.590, *P* = 0.023). Thus, rFA was the only diffusion variable included in the hierarchical regression analyses with the rest of the variables.

Before performing the regression analysis, we performed a correlation analysis between the possible predictors to determine the independence of the variables. CS _initial_, lesion load and lesion size were significantly correlated with the dependent variable (respectively, *r* = 0.810, *P* <0.001; *r* = −0.515, *P* = 0.02; −0.628, *P* = 0.003; see Supplementary Table 1).

After this, regression analyses were performed. First, we decided to run univariate regressions to determine the possible predictive power of each of the lesion-related measures, i.e., lesion size, lesion load of AF, and the initial severity (CS_initial_) on the CS_subacute_. Then, two hierarchical multivariate regressions were computed, using initial severity and age as covariates in the first block, but each one with a different variable that represented the lesion measures in the second block: the first regression included the lesion load of the left AF in the second block; in the second regression, we used the lesion size instead of the lesion load. Results are reported in Table 3. Regressions with lesion size, lesion load, and initial severity were found to be significant, accounting, respectively, for 39, 26.5, and 67.3% of the variance of the dependent variable. The next step consisted of performing a multivariate regression analyses with the previous variables and age as a covariate. When combined in the first block of the hierarchical analysis, CS _initial_ and age explained 67.8% of the variance (*R*^2^ = 0.678), with a *F* = 17.874 (*P* <0.001, *df* = 19), and CS _initial_ being the only variable whose beta coefficient was significant (β = 0.824; *P* <0.001). Adding the second block to the model allowed us to see two possible results that depended on the lesion-related variable. If lesion load was added, it did not change the *R*^2^, and the CS_initial_ was still the only significant coefficient (*P* = 0.001). If lesion size was added, it explained up to 71.7% of the variance (*R*^2^ = 0.717) with a *F* = 10.130 (*P* <0.001, *df* = 19). We added a third block in each regression, which included the rFA. Inclusion of this variable increased 2.6% in the variance account of the regression that used the lesion load (*R*^2^ change = −0.007), and 2.3% in the case of the regression that used lesion size (*R*^2^ change = −0.006). Both changes were not significant. We decided to run a variance inflation factor analysis (VIF) to discard multi-collinearity among the predictors, since two of these predictors in each model were highly correlated with the dependent variable. Collinearity was not significant in the present analysis (VIFs <2; see Table 3).

**Table 3:** Summary of results from regression models using CS_subacute_ as dependent variable.

**Model**	**Independent variables**	**ANOVA F (P)**	**R^**2**^**	**Beta standardized coefficients (B, P)**	**VIF**
Backwards	FA_r_ + AD_L_	3.49 (0.05)	0.3	rFA (**0.571, 0.023**)^*^	rFA (1.259) AD_L_ (1.259)
Univariate	CS_initial_	37.17 (<0.001) ^**^	0.673		
Univariate	Lesion size	11.75 (0.003)^*^	0.39		
Univariate	Lesion load	6.506 (0.02)^*^	0.265		
Hierarchical	Age, CS_initial_, Lesion size, rFA	10.130 (<0.001)^**^	0.73	CS_initial_ (0.659, 0.001^**^) Age (0.056, 0.736) Lesion size (-0.217, 0.240) rFA (0.136, 0.420)	CS_initial_ (1.563) Age (1.479) Lesion size (1.74) rFA (1.454)
Hierarchical	Age, CS_initial_, Lesion load, rFA	9.036 (0.001)^**^	0.703	CS_initial_ (0.789, 0.001^**^) Age (0.137, 0.394) Lesion load (0.31, 0.881) rFA (0.188, 0.273)	CS_initial_ (1.563) Age (1.479) Lesion load (1.74) rFA (1.454)

* *P <0 .05*.

** *P <0.001*.

*rFA, right Fractional Anisotropy from right arcuate fasciculus; ADl, Axial Diffusivity from left arcuate Fasciculus; CSinitial, Composite Score at initial assessment*.

*Significant predictors are highlighted in bold*.

## Discussion

Substantial improvement in language performance occurred within the first 2 weeks after stroke; this was measured using a composite score of several language functions in patients with mild to severe aphasia. As previously reported, there was a significant correlation between the degree of the achieved recovery and the potential improvement; however, our assessment time points were different than those previously reported in a study using similar measures (13). As for the predictions of the composite score during the early sub-acute phase, the most successful model consisted of a combination of age, lesion size, initial aphasia severity, and FA of the long segment of the right AF. Even without the diffusion measure, the model could predict up to 70% of the variance of the severity during the sub-acute phase. Most importantly, the predictive power of the initial aphasia severity (univariate model) was close to the multivariate models including lesion measures, which indicates that among all our variables, it was the best predictor for severity at the second time point.

Recovery from aphasia peaks during the first weeks after onset (3, 4) but it is difficult to ensure that all changes in the abilities are constrained by time. We have reported here, as has also been recently reported elsewhere (20), that it is possible to capture this process with a sensitive and reliable assessment. As it is typical when quantifying these processes, patients with higher initial severities also show more recovery, due to a larger space for possible improvement. Other patients with a lower initial severity improved less, or even slightly deteriorated during this period. These patients' recovery results may depend on other factors that do not systematically contribute to their recovery as successfully as in other patients. The reasons for this may vary among individuals, from the brain's blood supply and modulation of post-stroke neuroinflammation (5) to factors such as previous language use or socio-individual situation.

Most studies have investigated the prediction of language performance (for long term outcomes) such that the “size or site,” or any combination of both, could explain severity, symptoms and prognosis of aphasia (28, 31, 32, 34, 50). Conversely, we present evidence that different factors may account for the early phases of recovery, and more specifically, influence the spontaneous recovery. Previous studies have reported that initial aphasia severity, isolated or in combination with other biological measures, can account for a large amount of variance in the long term (13, 17). It has been also shown that different white matter structures may be involved in the outcome of aphasia at different stages, although this has not been explored during early recovery (50). Based on previous evidence on long term outcomes and the present data on subacute outcomes, we hypothesize that initial language severity may have a greater influence for short-term overall language prediction while lesion-related variables, though being correlated with early outcomes, possibly have a more important role in prediction of later phases of recovery, although this remains to be studied.

In our initial hypothesis, based on previous studies (30, 34, 37), we predicted that both the left and the right arcuate fasciculus would be related to improvement in language outcomes. One of the main hypotheses about the mechanisms of aphasia recovery is the involvement of spared contralateral homolog structures during the acute phase, as a prelude to a different stage of recovery where left hemisphere structures are involved (33, 50) reflecting a better long-term recovery. However, its involvement, as measured using FA, is much less significant when introduced into a multivariate model. One explanation is that recovery process has not yet reached its peak of stability because pathophysiological processes may have avoided a right “uptake” from the right arcuate fasciculus, and the timing of the assessment may have been too close to stroke onset to see differences. FA may also not be the best diffusion measure to characterize white matter in this stage, which should be investigated in comparison to other measures in future research. Although growing evidence has highlighted the structural integrity of the left arcuate fasciculus as a predictor of language performance in chronic phases of aphasia, the present results suggest that it does not account for early post-stroke aphasia outcomes. Our results suggest that only the right arcuate fasciculus predicts better aphasia outcomes after stroke in the acute/subacute phase, in line with the results reported by Forkel et al. (34).

Limitations of this study include the small sample size and the analysis that was limited to only one white matter tract. Other structures that have been flagged as potential scaffolding for later recovery, such as the inferior fronto-occipital fasciculus or the uncinate fasciculus (36), should also be addressed to analyze this complex process. However, a large part of language outcome after almost 2 weeks in individuals with aphasia has been explained using linguistic assessments and lesion measures. This suggests that cognitive evaluation remains as a powerful tool in the acute stages of aphasia and in the study of its evolution. Another limitation of the present study is the lack of quantitative measures of bilingualism which have been associated with the degree of aphasia recovery (e.g., age of acquisition, language use, etc.). Nonetheless, to minimize its effect on the present results, we tested patients in their dominant language and only recruited patients who had as dominant language one of the two broadly spoken languages in Quebec. Lastly, some authors have highlighted the possible inflation of recovery measures in prediction models of aphasia outcome, as well as in other post-stroke impairments (51–53). Our analyses only use outcome measures, which results in less possible mathematical coupling and therefore more straightforward interpretations.

In conclusion, future studies should address differences between recovery phases with more neuroimaging measures and with a larger sample to help account for the variability that post-stroke aphasia presents in daily clinical practice.

## Data Availability Statement

The datasets generated for this study are available on request to the corresponding author.

## Ethics Statement

The studies involving human participants were reviewed and approved by Research center of the Centre intégré universitaire de santé et de services du Nord-de-l'Île-de-Montréal, in the Hôpital Sacré Coeur de Montreal (Université de Montréal). The patients/participants provided their written informed consent to participate in this study. Written informed consent was obtained from the participants for any potentially identifiable human images or data is presented in this study.

## Author Contributions

AO prepared and analyzed the data, interpreted the results, and wrote the manuscript. SB and KM developed the study, interpreted the results, and reviewed the manuscript. AB and MD-B recruited the participants, collected the data, collaborated in the data preparation, and reviewed the manuscript. CB and BH worked on the data processing, data preparation, and reviewed the manuscript. ER and CL participated in the elaboration of the study, and reviewed the manuscript. AD collaborated in the elaboration of the study, in the recruitment and reviewed the manuscript.

### Conflict of Interest

AD received research grants from Flamel Ireland, Canopy Growth, Jazz Pharma, Biron and served on advisory boards for Eisai, Jazz Pharma and UCB. The remaining authors declare that the research was conducted in the absence of any commercial or financial relationships that could be construed as a potential conflict of interest.

## Supplementary Material

Supplementary Figure 1 Differences between overall severity and subscores in acute and subacute phase assessments (significance set at á = 0.05).

Supplementary Figure 2 Visualization of the relation between subacute aphasia severity (CSsubacute) and the Predicted values for each participant using the hierarchical model with Lesion size as lesion-related measure.

Supplementary Table 1 Correlation Matrix. Matrix with all correlations between independent variables (Initial severity, Age, Lesion load, Lesion size, rFA) and the dependent variable (CS_*subacute*_). Pearson coefficients are reported (level of P). All correlations have been corrected to a threshold of α = 0.01.
