# Supplementary figures and images for: Predicting Early Post-stroke Aphasia Outcome From Initial Aphasia Severity
Source: Front Neurol. 2020 Feb 21;11:120. doi: 10.3389/fneur.2020.00120 (PMC7047164; doi:10.3389/fneur.2020.00120)

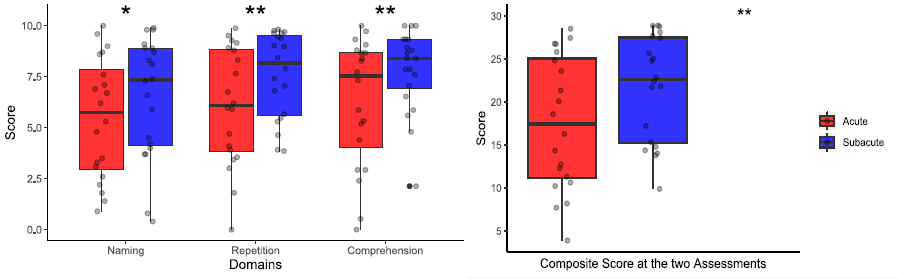

Supplement: Supplementary Figure 1 — Differences between overall severity and subscores in acute and subacute phase assessments (significance set at á = 0.05). [file Image_1.TIF]

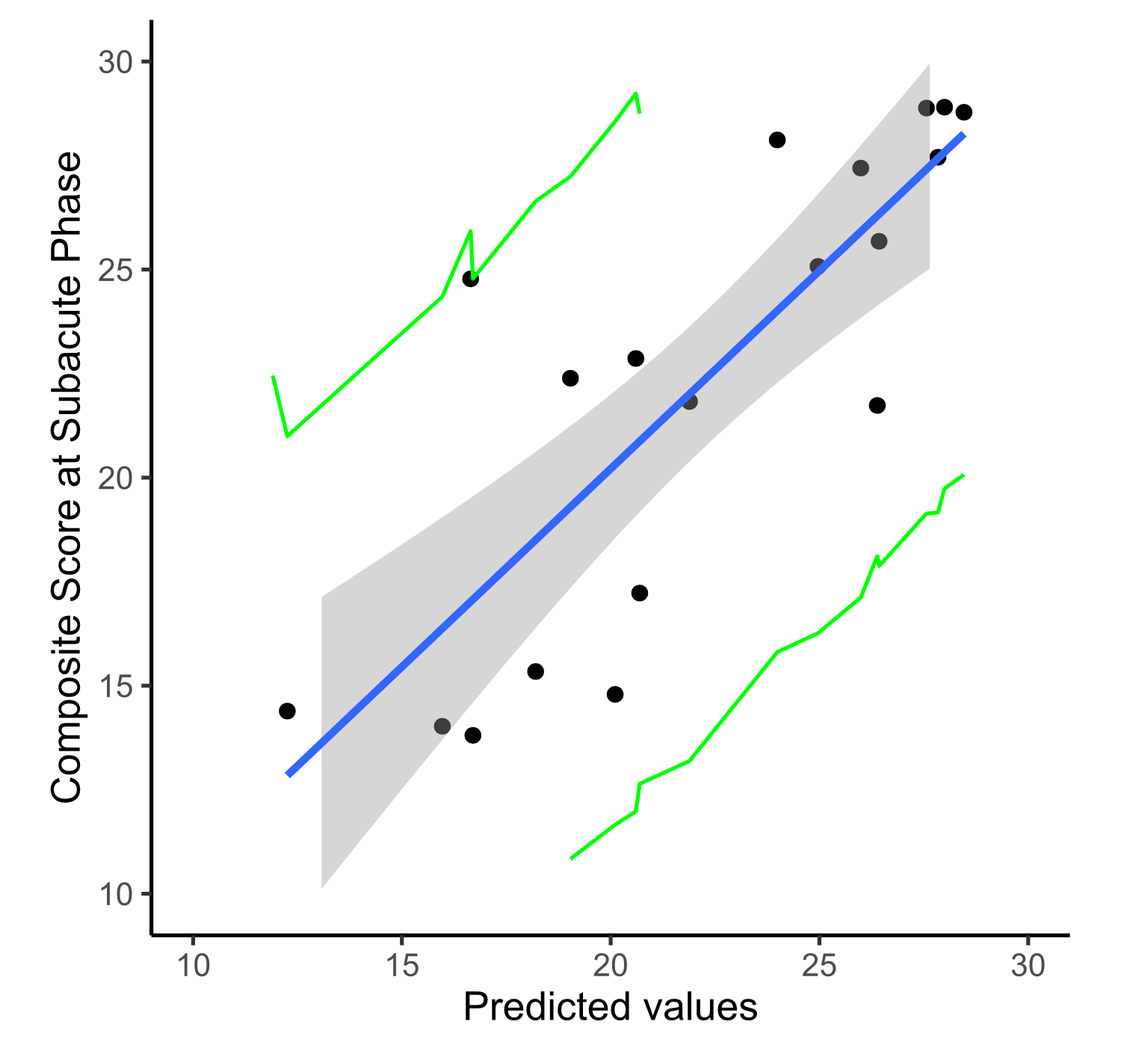

Supplement: Supplementary Figure 2 — Visualization of the relation between subacute aphasia severity (CSsubacute) and the Predicted values for each participant using the hierarchical model with Lesion size as lesion-related measure. [file Image_2.TIF]
