# Supplementary material for: Predicting Early Post-stroke Aphasia Outcome From Initial Aphasia Severity
Source: Front Neurol. 2020 Feb 21;11:120. doi: 10.3389/fneur.2020.00120 (PMC7047164; doi:10.3389/fneur.2020.00120)
Supplement: Supplementary Table 1 — Correlation Matrix. Matrix with all correlations between independent variables (Initial severity, Age, Lesion load, Lesion size, rFA) and the dependent variable (CSsubacute). Pearson coefficients are reported (level of P). All correlations have been corrected to a threshold of α = 0.01. [file Table_1.pdf]

Supplementary Material

Supplementary Table 1. Correlation Matrix. Matrix with all correlations between independent variables (Initial severity, Age, Lesion load, Lesion size, rFA) and the dependent variable ($CS_{subacute}$). Pearson coefficients are reported (level of P). All correlations have been corrected to a threshold of α = 0.01.

	**Initial severity ($CS_{initial}$)**	**Age**	**Lesion load**	**Lesion size**	**rFA**	**$CS_{subacute}$**
**Initial severity**	________	-0.045 (0.850)	-0.712 (<0.001)**	-0.555 (.011)*	0.333 (0.151)	0.821 (<0.001)**
**Age**		______	0.006 (0.980)	-0.335 (0.189)	-0.417 (0.097)	0.051 (0.846)
**Lesion load**			_______	0.457 (0.065)	-0.163 (0.533)	-0.571 (0.009)*
**Lesion size**				______	-0.335 (0.189)	-0.625 (0.003)*
**rFA**					_______	0.388 (0.09)

* P < 0.01

** P < 0.001

rFA = Fractional Anisotropy from right arcuate fasciculus
