# Supplementary material for: Predicting Early Post-stroke Aphasia Outcome From Initial Aphasia Severity
Source: Front Neurol. 2020 Feb 21;11:120. doi: 10.3389/fneur.2020.00120 (PMC7047164; doi:10.3389/fneur.2020.00120)
Supplement: Supplementary file 4 [file Data_Sheet_1.pdf]

Supplementary Material

1. Description of the composite score derived from standardized subtests

1. Language comprehension subscore

Tasks:
- Word-sentence comprehension task of *MT-86-Protocole Montréal-Toulouse d'examen linguistique de l'aphasie* (39) (maximum score of 47 points)
- Sequential commands of the revised (short) version of the Token Test (40) (maximum score of 36 points)

Subscore calculation (maximum score of 10):
((Raw score word-sentence comprehension task + Raw score sequential commands) *10)/83

2. Repetition subscore

Tasks:
- Word and nonword repetition task of *MT-86-Protocole Montréal-Toulouse d'examen linguistique de l'aphasie* (39) (maximum score of 30)
- Sentence repetition task of the *MT-86-Protocole Montréal-Toulouse d'examen linguistique de l'aphasie* (39) (maximum score of 5)

Subscore calculation (maximum score of 10)
((Raw score word and nonword repetition*2 + Raw score sentence repetition*5)*10)/75

3. Naming subscores

3.1 Naming subscore for French speaking participants

Tasks:
- Picture naming task from the *Test de Denomination Orale d'images* (DO-80) (42) (maximum score of 60 points)
- Semantic fluency task of the *Protocole Montréal d'Évaluation de la Communication* (41) (maximum score set at 25 points)

Subscore calculation (maximum score of 10):
((Raw score picture naming + Raw score semantic fluency)*10)/105

3.2 Naming subscore for English speaking participants

Tasks:
- Picture naming task of the Boston Naming Test (38) (maximum score of 60 points)
- Semantic fluency task of the *Protocole Montréal d'Évaluation de la Communication* (41) (maximum score set at 25 points)

Subscore calculation (maximum score of 10):
((Raw score picture naming + Raw score semantic fluency)*10)/85

References (as cited in the article):

38. Goodglass H, Kaplan E, Barresi B, Goodglass H, Goodglass H, Goodglass H, et al. The Boston Diagnostic Aphasia Examination : BDAE-3 long form kit. (2001) Philadelphia: Lippincott Williams & Wilkins.

39. Nespoulous JL, Lecours AR, Lafond D, Lemay MA, Puel M, Joanette Y, et al. Protocole Montréal-Toulouse d'examen linguistique de l'aphasie: MT-86 module standard initial, M1b(2e édition révisée par Renée Béland et Francine Giroux). Isbergues, France: Ortho Edition; 1992.

40. De Renzi E, Faglioni P. Normative data and screening power of a shortened version of the Token Test. Cortex [Internet]. 1978;14(1):41–9.

41. Joanette Y, Ska B, Côté H. Protocole Montréal d'Évaluation de la Communication. Isbergues, France: Ortho Édition; 2004.

42. Deloche G, Hannequin D. Test de dénomination orale d'images: DO-80. Éditions du centre de psychologie appliquée; 1997.
